# 

**Published:** 1887-08-13

**Authors:** 


### CONTENTS.

**Unmarried Women** - - - - - - 325

**Words of Consolation.**—XLV. The Sufferings of Christ - - - - - - - - 326

**Religio Medici** - - - - - - - 327

**The National Pension Fund** - - - - - 328

**Guild of Friends of the Infirm in Mind.** By the Rev. H. Hawkins, Chaplain, Colney Hatch - 329

**The Doctor's Pulpit:** Hypochondriasis - - 331

**Notes and News.**—Competitive Designs for Hospitals.—Hospital Annual Reports.—The Bute Hospital.—Guy's Hospital and the Special Appeal Fund.—Vacancies.—Beach Rock Convalescent Home.—The Lowestoft Convalescent Home.—Demonstration in Aid of the Huddersfield Infirmary.—The Liverpool Royal Infirmary and its Officials.—Proposed New Special Hospital for Nottingham, etc., etc. - - - - - 332

**Annotations.**—Costly Patients.—Where is the Colchester Physician?—Doctors in Council.—Unparalleled Scepticism - - - - - - - - 335

**Floating Hospital on the River Tyne** - - 336

**Chats about New Books:** Marrying and Giving in Marriage - - - - - - - 337

**Presentation to Mr. P. Michelli** - - - 337

**Till the Doctor Comes.**—XXIV. Immediate Treatment in Cases of the more Common Poisons - 338

**Cruel Treatment of a Female Patient by her Parents** - - - - - - 339

**Invalid Children** - - - - - - 339

**Poetry:** The Stethoscope Song. By Oliver Wendell Holmes - - - - - - - - 340

**The Children's Corner.**—Puzzles, Answers, etc. - vi

**Amusements with Profit** - - - - - vi

